# Self-preservation and structural transition of gas hydrates during dissociation below the ice point: an *in situ* study using Raman spectroscopy

**DOI:** 10.1038/srep38855

**Published:** 2016-12-12

**Authors:** Jin-Rong Zhong, Xin-Yang Zeng, Feng-He Zhou, Qi-Dong Ran, Chang-Yu Sun, Rui-Qin Zhong, Lan-Ying Yang, Guang-Jin Chen, Carolyn A. Koh

**Affiliations:** 1 State Key Laboratory of Heavy Oil Processing, China University of Petroleum, Beijing, 102249, China; 2 Center for Hydrate Research, Colorado School of Mines, Golden, Colorado 80401, United States

## Abstract

The hydrate structure type and dissociation behavior for pure methane and methane-ethane hydrates at temperatures below the ice point and atmospheric pressure were investigated using *in situ* Raman spectroscopic analysis. The self-preservation effect of sI methane hydrate is significant at lower temperatures (268.15 to 270.15 K), as determined by the stable C-H region Raman peaks and *A*_L_/*A*_S_ value (Ratio of total peak area corresponding to occupancies of guest molecules in large cavities to small cavities) being around 3.0. However, it was reduced at higher temperatures (271.15 K and 272.15 K), as shown from the dramatic change in Raman spectra and fluctuations in *A*_L_/*A*_S_ values. The self-preservation effect for methane-ethane double hydrate is observed at temperatures lower than 271.15 K. The structure transition from sI to sII occurred during the methane-ethane hydrate decomposition process, which was clearly identified by the shift in peak positions and the change in relative peak intensities at temperatures from 269.15 K to 271.15 K. Further investigation shows that the selectivity for self-preservation of methane over ethane leads to the structure transition; this kind of selectivity increases with decreasing temperature. This work provides new insight into the kinetic behavior of hydrate dissociation below the ice point.

Gas hydrates are nonstoichiometric crystalline solids containing guest molecules within the cages formed by host hydrogen-bonded water molecules at low temperature and high pressure conditions^1^. Three common clathrate hydrate structures have been identified, cubic structure I (sI), cubic structure II (sII) and hexagonal structure H (sH). These structures are comprised of polyhedral water cages, which can trap different guest (gas) molecules. Specifically in each repeating unit crystal, sI contains two pentagonal dodecahedral cavities (12 pentagonal faces, 5^12^) + six tetrakaidecahedral cavities (12 pentagonal and 2 hexagonal faces, 5^12^6^2^); sII contains sixteen 5^12^ cavities + eight hexakaidecahedral cavities (12 pentagonal and 4 hexagonal faces, 5^12^6^4^); sH contains three 5^12^ cavities + two dodecahedral cavities (three square, six pentagonal, and three hexagonal faces, 4^3^5^6^6^3^) + one icosahedral cavity (12 pentagonal and 8 hexagonal faces, 5^12^6^8^)^1^,^2^. Gas hydrates have drawn attention in the natural gas and oil industries since the 1930s as a consequence of the finding that hydrate formation may lead to plugging of gas pipelines^3^,^4^,^5^,^6^,^7^. Recently, hydrates have been of increasing interest, mainly because of the substantial amounts of natural gas found in hydrate deposits in oceanic and arctic sediments, and are considered as a potential new energy resource^8^,^9^,^10^,^11^,^12^,^13^. Additionally, several researchers have investigated the development of new technologies based on gas hydrates, *e.g.*, separation of gas mixtures, and storage of natural gas, H_2_, or CO_2_^14^,^15^,^16^,^17^,^18^,^19^,^20^,^21^. The mechanism(s) of hydrate formation and dissociation are of fundamental importance to the different applications of gas hydrates^22^. Fundamental mechanistic insight requires knowledge of the structure and structural changes/transitions during hydrate formation and dissociation. More importantly, the hydrate structural properties have a significant impact on the storage capacity and the stability of hydrates^23^,^24^,^25^,^26^,^27^,^28^.

The structure of a hydrate depends on not only the composition of hydrate formers, but also temperature and pressure conditions^15^,^29^,^30^,^31^,^32^,^33^,^34^. The structural transition(s) of hydrates, which can occur by changing composition and temperature/pressure conditions have been previously reported^35^,^36^,^37^,^38^,^39^. For example, methane forms sI hydrate under moderate conditions. However, it transforms to sII hydrate at 100 MPa, and then to a ‘sH’ type hydrate at 600 MPa^40^. Gas mixtures of methane and ethane, with methane content lower than 75 mol.% or higher than 99 mol.%, form sI hydrates. Otherwise, they form sII hydrate^41^,^42^. Up to now, most structural transition studies have been performed during the formation process of hydrates^37^,^43^,^44^, while there are limited studies focused on the dissociation process^43^,^45^. In the latter dissociation studies, the conditions have been mostly outside/not focused on the hydrate self-preservation region. However, the kinetic behavior and stability of hydrates during dissociation are critical to the different applications of hydrates, such as gas storage (methane, hydrogen, carbon dioxide, *etc.*) and exploitation of naturally occurring hydrate deposits. The study of hydrate dissociation from a physicochemical point of view will also increase understanding of the chemistry of water and hydrate related climate change on Earth^2^.

A series of macroscopic investigations of hydrate dissociation kinetics have been performed in recent decades^46^,^47^,^48^,^49^. Some abnormal phenomena were observed in these studies, *i.e.*, the self-preservation effect of hydrates below the ice point^50^,^51^,^52^,^53^,^54^,^55^,^56^,^57^,^58^,^59^,^60^,^61^, or reformation of hydrates during the production of gas from naturally occurring hydrate deposits by depressurization^62^. Some hydrates, such as methane hydrate, exhibit abnormal stability at around 268.15 K, in which they can remain stable for several days at atmospheric pressure^63^. Below or above this temperature, the stability will decrease^50^. C_2_H_6_ and C_3_H_8_ hydrates were considered to show no preservation phenomenon, and a mixture of C_2_H_6_ or C_3_H_8_ with CH_4_ reduces the preservation ability of CH_4_ hydrate^53^,^64^,^65^. For hydrate formed from (82 mol.% CH_4_ + 18 mol.% C_2_H_6_) gas mixture, no comparable preservation effect was exhibited when rapidly depressurized at 268 K^64^. However, there are also reports that pure ethane hydrate and methane–ethane mixed gas hydrates can exhibit a self-preservation effect^66^. The self-preservation effect is critical for the feasibility of hydrate-based gas storage technologies. To-date, the mechanism of these abnormal phenomena is still unclear. Therefore, new insight into the mechanism of self-preservation can be provided by molecular investigations of the structural transition(s) during hydrate decomposition.

Raman spectroscopy is a powerful and convenient tool (with fast and relatively simple *in-situ* sampling) to determine the structure and structure transition(s) of hydrates^67^,^68^. In this work, *in situ* Raman spectroscopy was employed to study the dissociation behavior of pure methane hydrate and binary methane-ethane double hydrate at temperatures below the ice point and atmospheric pressure. The new molecular observations from this work could help to provide new insight into the dissociation kinetic behavior of hydrates below the ice point.

## Results

Five groups of methane hydrate samples and five groups of methane-ethane double hydrate samples were formed in a high-pressure optical cell (HPOC) comprising a sapphire window, using pure methane with an aqueous solution of 1000 mg/L of sodium dodecyl sulfate (SDS) at 276.15 K and 5.0 MPa, or a gas mixture containing 68 mol.% methane (C1) and 32 mol.% ethane (C2) with deionized water at 276.15 K and 3.0 MPa. A more detailed description of the experimental apparatus is given in the *Methods* section. Prior to the dissociation of each hydrate, the system was cooled to a designated temperature and then held for 2 hours allowing the hydrate to equilibrate with the gas phase at this temperature. After this, the hydrate was slowly depressurized to the equilibrium pressure corresponding to the set temperature, and then quickly depressurized to atmospheric pressure in the HPOC cell. From that moment, time-dependent measurements of hydrate dissociation using *in situ* Raman spectroscopy were started. The hydrate structure and composition were determined from the C-H region Raman band frequencies, corresponding to the vibrational stretching modes of methane and ethane molecules in the large and small cavities of sI and sII hydrate lattices (summarized in Table 1). These frequencies were determined based on the experimental results of Ohno *et al*.^69^ and Subramanian *et al*.^38^,^44^, and our Raman measurements on the pure methane hydrates and methane-ethane double hydrates. Additionally, it is known that the gas phase methane molecule has a C-H stretching mode at around 2915 cm^−1^, and gas phase ethane has a C-H stretching mode at around 2899 cm^−1^ and 2953 cm^−1 ^38,44.

### Pure methane hydrate

**Figure 1:**
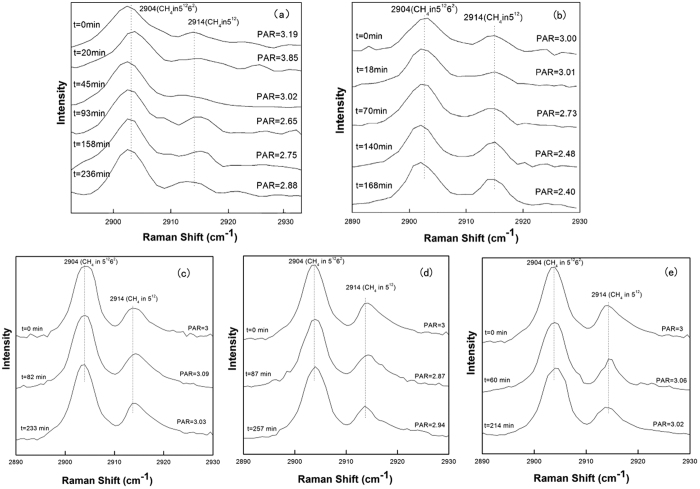
Raman spectra of the C-H region for methane hydrate obtained at different times during hydrate dissociation at (**a**) 272.15 K, (**b**) 271.15 K, (**c**) 270.15 K, (**d**) 269.15 K, and (**e**) 268.15 K respectively and atmospheric pressure.

Figure 1 shows the representative Raman spectra for the C-H stretching mode of methane hydrate during the dissociation process at temperatures of 272.15 K, 271.15 K, 270.15 K, 269.15 K and 268.15 K and atmospheric pressure. The Raman spectra collected at *t* = 0 min denote the re-equilibrium state of methane hydrate at the related temperature, before the rapid depressurzation of the system to atmospheric pressure. Under those conditions, all Raman spectra measured at five temperatures show two peaks centered at 2904 cm^−1^ and 2914 cm^−1^, which correspond to C-H stretching frequencies of methane in large (2904 cm^−1^) and small (2914 cm^−1^) cavities of sI hydrate. The Raman spectra of methane hydrate collected during the dissociation process within about 4 hours at each temperture show that there is no change in peak positions with time, suggesting that the structure type (sI) of methane hydrate remains unchanged. However, obvious changes in the relative intensities of the two C-H peaks with the elapsed decomposition time are observed at 272.15 K and 271.15 K, respectively, especially for the peak at 2914 cm^−1^. However, changes were insignificant for cases at lower temperatures (270.15 K, 269.15 K and 268.15 K). In order to show this phenomenon more clearly, Fig. 1 also shows the peak area ratios (PAR) values for methane hydrate at different temperatures and times, *i.e.,* the peak area ratios of CH_4_ molecules in large cavities (5^12^6^2^) to small cavities (5^12^), *A*_L_/*A*_S_. The PAR values of the two C-H peaks at 272.15 K show an unexpected trend of decrease-increase-decrease. However, at lower decomposition temperatures (≤270.15 K), the PAR values of the two peaks remain above around 3, which is consistent with 3:1 large to small cavity ratio of sI hydrate, indicating the self-preservation effect of methane hydrate is significant at lower decomposition temperatures (≤270.15 K), but not at higher temperatures (≥271.15 K). This latter concept is in close agreement with the macroscopic study by Stern *et al*.^64^, which indicated the self-preservation phenomenon occurred at lower temperatures.

### Methane-ethane double hydrate

**Figure 2:**
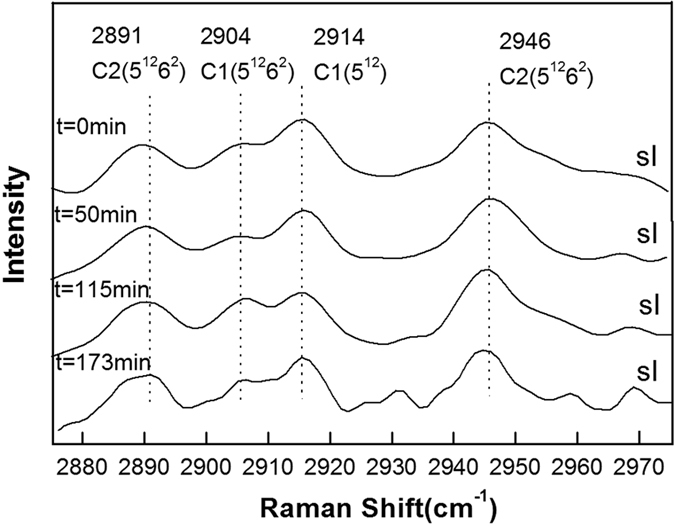
Raman spectra of the C-H region for methane-ethane double hydrate obtained at different times during hydrate dissociation at 272.15 K and atmospheric pressure. It should be noted that a large amount of spectra were collected on different locations of the hydrate sample. Only representative spectra are given in the figure to show the variation of Raman spectra with time, which also applies to Figs 3, 4, 5, 6.

**Figure 3:**
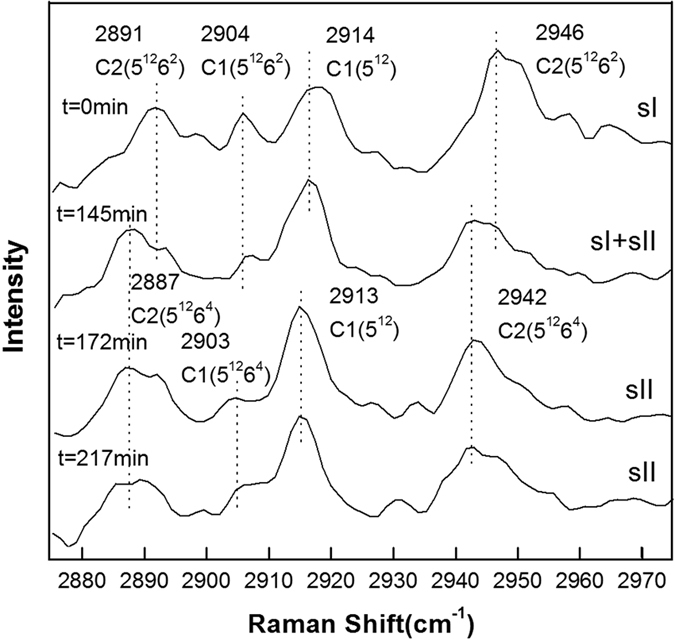
Raman spectra of the C-H region for methane-ethane hydrate obtained at different times during hydrate dissociation at 271.15 K and atmospheric pressure.

**Figure 4:**
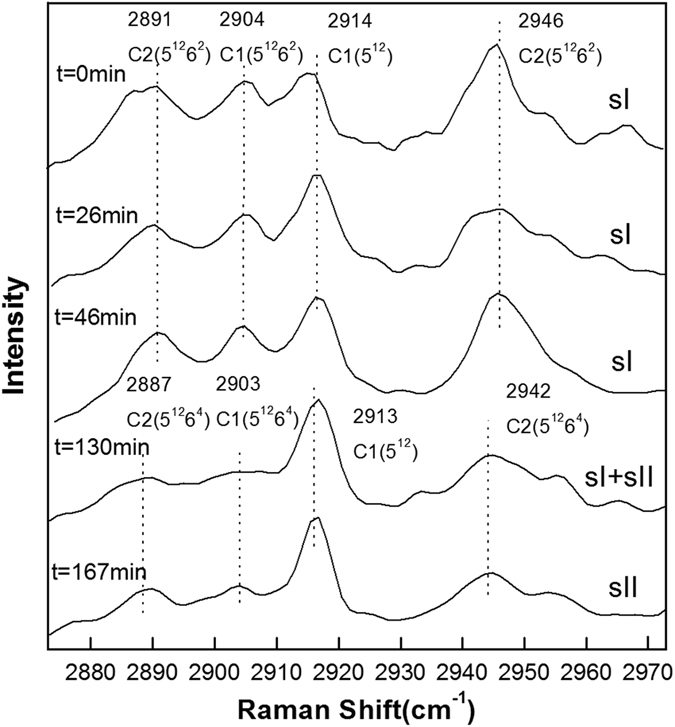
Raman spectra of the C-H region for methane-ethane hydrate obtained at different times during hydrate dissociation at 270.15 K and atmospheric pressure (The structure transition from sI to sII occurred at around 93 min).

**Figure 5:**
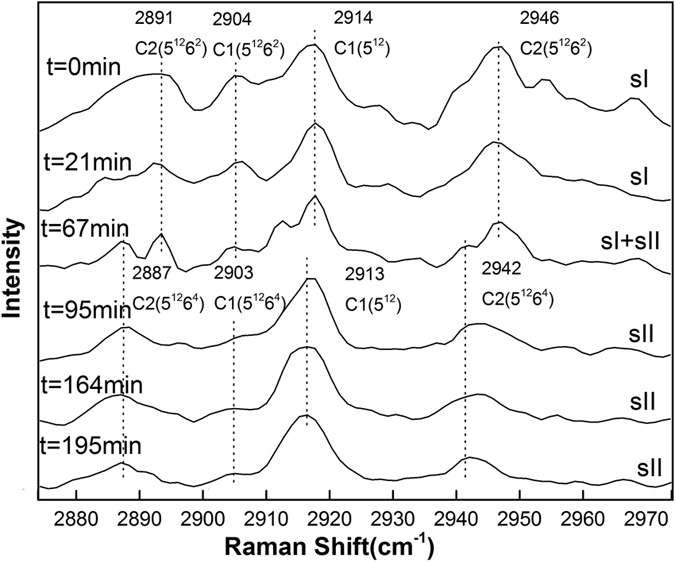
Raman spectra of the C-H region for methane-ethane hydrate obtained at different times during hydrate dissociation at 269.15 K and atmospheric pressure.

**Figure 6:**
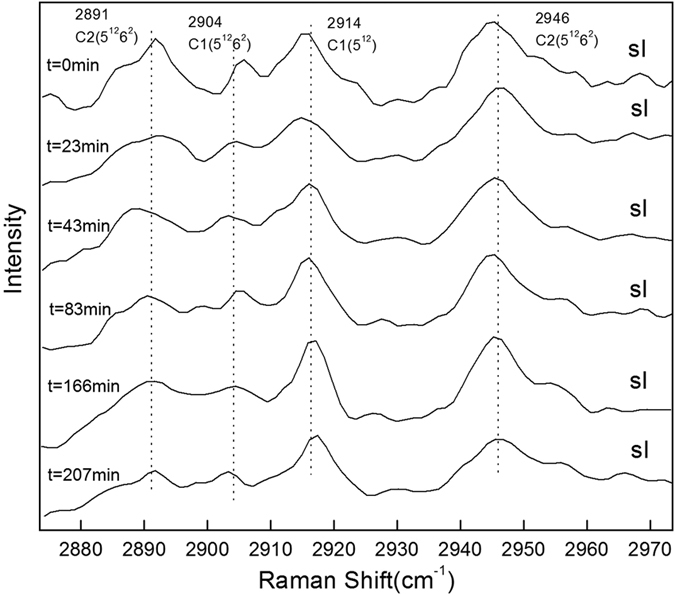
Raman spectra of the C-H region for methane-ethane hydrate obtained at different times during hydrate dissociation at 268.15 K and atmospheric pressure.

Figures 2, 3, 4, 5, 6 show the representative Raman spectra for the C-H stretching modes of methane-ethane double hydrates with the elapsed decomposition time at 272.15 K, 271.15 K, 270.15 K, 269.15 K, and 268.15 K and atmospheric pressure. The Raman spectra collected at *t* = 0 min at all five temperatures show the same four peaks. Amongst them, two peaks centered at 2891 and 2946 cm^−1^ could be assigned to C-H stretching frequencies of ethane trapped in large (5^12^6^2^) cages of sI hydrate, and the other two centered at 2904 and 2914 cm^−1^ could be assigned to the C-H stretching frequencies of methane trapped in large (5^12^6^2^) and small (5^12^) cages of sI hydrate, respectively. Furthermore, the PAR values corresponding to the molar ratio of guest molecules trapped in large cavities to those in small cavities, *A*_L_/*A*_S_, were determined to be around 3, which is consistent with the 3:1 large to small cavity ratio in the sI hydrate unit cell. Thus, it could be deduced that the gas mixture containing 68 mol.% CH_4_ + 32 mol.% C_2_H_6_ forms sI hydrate at temperatures ranging from 268.15 to 272.15 K at approximately 3.0 MPa, which is also in agreement with CSMGem^1^ and Takeya *et al*.^39^. During the decomposition of this double hydrate at 272.15 K and 268.15 K, there is no significant change in peak positions with time as shown in Figs 2 and 6, suggesting that the structure type (sI) of methane-ethane double hydrate remains unchanged. However, it is interesting that the structure transition from sI to sII was observed during the decomposition of this double hydrate at 271.15 K, 270.15 K, and 269. 15 K. With the elapsed decomposition time, four new peaks appear close to the four original peaks as shown in Figs 3, 4, 5. Two new peaks centered at 2887 and 2942 cm^−1^ could be assigned to the C-H stretching frequencies of ethane trapped in large (5^12^6^4^) cavities of sII hydrate, and the other two centered at 2903 and 2913 cm^−1^ could be assigned to the C-H stretching frequencies of methane trapped in large (5^12^6^4^) and small (5^12^) cavities of sII hydrate, respectively. The coexistence of the new and original peaks indicates the coexistence of the sI and sII hydrate structures. Gradually, the peaks corresponding to the sI hydrate disappeared, and the structure entirely transformed into sII hydrate.

The structure transition is displayed by not only the shift in the C-H peak positions, but also by the change in relative intensities of the C-H peaks. The number of large cavities (5^12^6^2^) for sI hydrate is three times that of small ones (5^12^), i.e. the PAR values are about 3:1 for methane guest molecules. This characteristic is observed for Raman spectra of hydrates before decomposition or at the initial stage of decomposition. After the structure transition, the PAR value decreases and tends to those of sII hydrate, which is the ratio of the number of large cavities (5^12^6^4^) to that of small ones (5^12^). By comparing Figs 3, 4, 5, one can see that the elapsed decomposition time increases with decreasing temperature, when hydrate structure transitions occur. The structure transition from sI to sII occurred at 145 min, 93 min, and 67 min at decomposition temperatures of 271.15 K, 270.15 K, and 269.15 K, respectively. These observations may be due to hydrate dissociation being retarded with increasing temperature during this temperature region.

## Discussion

*In situ* Raman spectroscopy can be used to evaluate on the molecular-level the self-preservation effect of methane hydrate and structural transition of methane-ethane hydrate during dissociation below the ice point at atmospheric pressure. Two types of the PAR values were determined, corresponding to the molar ratio of guest molecules trapped in large cavities to those in small cavities (*A*_L_/*A*_S_), and the molar ratio of methane to ethane molecules in binary methane-ethane hydrate (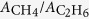). It should be noted that the peak centered at around 2916 cm^−1^ corresponds to the C-H stretching frequency of gas phase methane, and overlaps the peak centered at around 2914 cm^−1^ related to methane trapped in small (5^12^) cavities of sI hydrate, thus it was unavoidably included in the measurement of *A*_S_ and 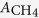.

### Pure methane hydrate

**Figure 7:**
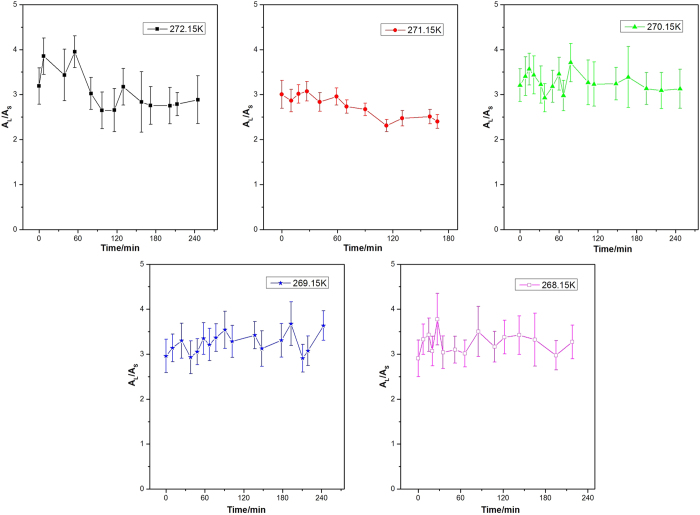
The variation of *A*_L_/*A*_S_ values with time for methane hydrate during dissociation at different temperatures.

Figure 7 shows the variation of the *A*_L_/*A*_S_ values for methane hydrate with time at different decomposition temperatures. Before dissociation, the *A*_L_/*A*_S_ value is about 3 at each temperature, which is consistent with the 3:1 large to small cavity ratio in the sI hydrate unit cell. Since all methane hydrates were formed at higher pressure (5.0 MPa), the occupancy percentage of methane molecules in both large and small cavities was assumed to be close to 100%, which is close to that predicted by CSMGem^1^. Hence, the ratio of guest molecules trapped in large and small cavities should be close to 3. As shown in Fig. 7, at higher decomposition temperatures of 272.15 K and 271.15 K, the *A*_L_/*A*_S_ values fluctuate dramatically with the elapsed time and tend to be lower than 3. The decrease of the *A*_L_/*A*_S_ value at the dissociation stage is caused by the sudden increase in the relative peak intensity at 2914 cm^−1^ (Fig. 1a at 93 min, and Fig. 1b at 70 min). This phenomenon could be related to the weak self-preservation effect of methane hydrate at these higher temperatures. In the beginning, the dissociation rate is high and a large amount of methane gas is released. As stated in the Results section, the C-H region band frequency for free methane gas is 2916 cm^−1^, which is very close to that for a methane molecule encaged in a small cavity, 2914 cm^−1^. That would lead to the overlap of two peaks related to C-H stretching frequencies in gas phase methane and methane encaged in small cavities of sI hydrate. This would therefore result in a sharp increase in peak intensity and peak area at 2914 cm^−1^ and corresponding decrease in *A*_L_/*A*_S_ value. On the other hand, the rapid release of methane gas from the hydrate would result in a distinct decrease in temperature (since hydrate dissociation is endothermic); thus the release of methane gas stops within a short time, and the *A*_L_/*A*_S_ value tends to increase. With the elapse of time, the temperature increases to the set value again, so the release of methane from hydrate restarts and the *A*_L_/*A*_S_ value decreases again. When the dissociation process tends to the end, the *A*_L_/*A*_S_ value also becomes stable.

Conversely, the *A*_L_/*A*_S_ value remains above 3 during the whole dissociation process at lower temperatures (270.15 K, 269.15 K and 268.15 K). This indicates the self-preservation effect is significant and the hydrate dissociation rate is very low within the examined time under these conditions, and results in the very slow release of methane from the hydrate, with little influence on the Raman spectra.

### Methane-ethane double hydrate

**Figure 8:**
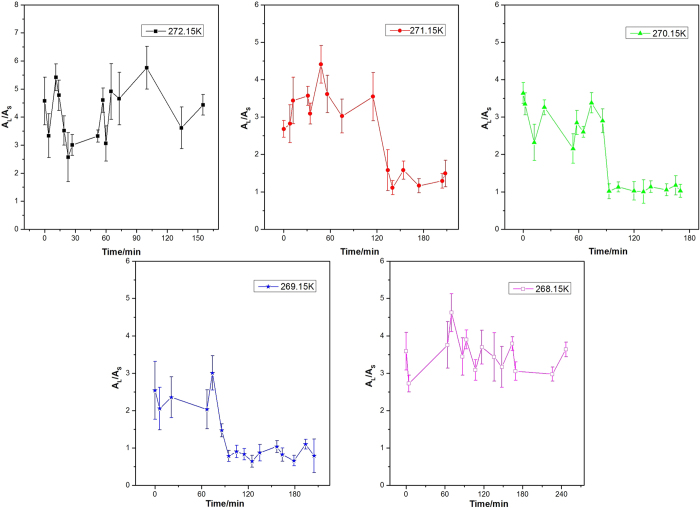
The variation of *A*_L_/*A*_S_ values with the elapsed decomposition time for methane-ethane double hydrate at different temperatures.

The varition of the *A*_L_/*A*_S_ value for methane-ethane double hydrate with the elapsed dissociation time at 272.15 K, 271.15 K, 270.15 K, 269.15 K, and 268.15 K and atmospheric pressure is illustrated in Fig. 8. *A*_L_ refers to the total area of peaks corresponding to the occupancies of methane and ethane molecules in large cavities (5^12^6^2^ for sI or 5^12^6^4^ for sII); *A*_S_ refers to the total area of peaks corresponding to the occupancies of methane and ethane molecules in small cavities (5^12^), where the latter is around zero. Just like the case for methane hydrate, the *A*_L_/*A*_S_ values for methane-ethane hydrate are around 3 before the dissociation at all five temperatures, which is in agreement with the relative intensities data of the various CH_4_ and C_2_H_6_ sites measured using ^13^C magic-angle spinning (MAS) NMR^45^. Similar to that for methane hydrate, the *A*_L_/*A*_S_ values of methane-ethane double hydrate also fluctuate at the early stage during the dissociation. Though the extent of fluctuation is more severe than methane hydrate. The reason may be that the dissociation rate of binary methane-ethane double hydrate is higher than that of pure methane hydrate at the same temperature and pressure, which is in agreement with Stern *et al*.^64^.

The most significant charateristic of the methane-ethane double hydrate dissociation behavior, which is different from pure methane hydrate, is the structural transition during hydrate dissociation at 271.15 K, 270.15 K, and 269.15 K. As observed at these three temperatures, the *A*_L_/*A*_S_ value is firstly maintained at around 3, and then decreases continuously and is finally stabilized at less than 1 after a certain period of decomposition time. Considering the 1:2 large to small cavity ratio and the representative peak positions of sII methane-ethane hydrate, this is consistent with the structural transition from sI to sII, which is also shown by Figs 3, 4, 5. Coversely, it can be seen from Fig. 8 that the *A*_L_/*A*_S_ value at 272.15 K fluctuates drastically with a range from approximately 3 to 5, but shows no obvious downtrend. The reason may be that the dissociation at higher temperature close to the ice point is significant. Therefore, obviates the occurrence of the structure transition for methane-ethane hydrate during the dissociation. In contrast, at lower temperatures such as 268.15 K as shown in Fig. 8, the *A*_L_/*A*_S_ value remains above 3 during the whole dissociation process. This indicates the self-preservation effect is more significant and the hydrate dissociation rate is very low within the examined time under this conditions, which results in the unchanged structure of hydrate and slow release of gas from the hydrate.

**Figure 9:**
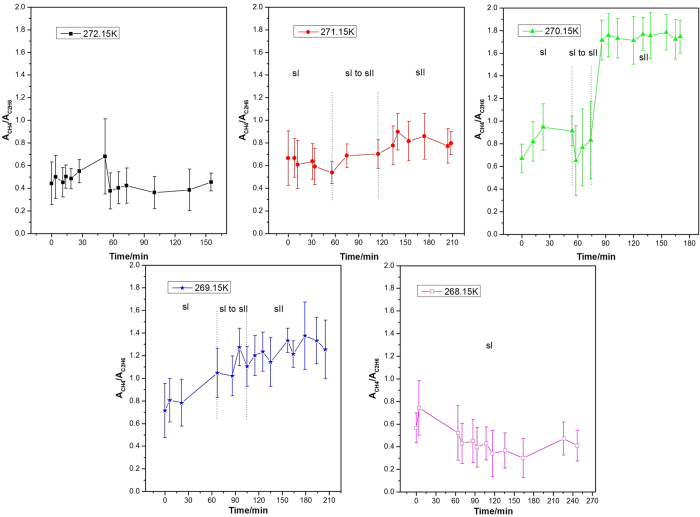
The variation of the 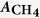/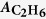 values with the elapsed decomposition time for methane-ethane double hydrate at different temperatures.

To further investigate the mechanism of the structural transition, another set of PAR values, 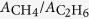, was calculated and compared, where 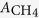 and 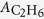 refer to the total area of peaks corresponding to the occupancies of methane molecules and that corresponding to the occupancies of ethane molecules in hydrate lattice respectively. Figure 9 shows the variation of the 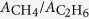 value with the elapsed decomposition time at each temperature for methane-ethane double hydrate. At higher temperature, e.g., 272.15 K, the 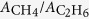 value remains at around 0.5 as shown in Fig. 9. Hydrate dissociates rapidly and ethane and methane gas molecules are rapidly released due to the weaker self-preservation effect at higher temperature. In contrast, when at a lower temperature, e.g., 268.15 K, the 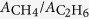 value tends to decrease during the dissociation process. The structure of methane-ethane double hydrate is maintained, but the reduced release rate of methane is higher than that of ethane from the hydrate cavities. (Here, the reduced release rate of methane or ethane is defined as the ratio of the release rate (mol/h) to original content of methane or ethane (mol) in the double hydrate). When at 271.15 K, 270.15 K, and 269.15 K, the dissociation process could be divided into three stages. The first stage is the single dissociation of sI hydrate, the second one is the structural transition from sI to sII, and the third one is the single dissociation of sII hydrate. The fluctuation of the 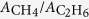 value in the first stage should be caused by the release of ethane and methane gas molecules. In the second stage, the 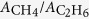 values increase gradually with the elapsed time at these three temperatures, demonstrating that ethane and methane molecules are not released in the same proportion to their original composition ratio in the sI hydrate lattice, *i.e.*, the reduced release rate of ethane is higher than that of methane from the hydrate cavities. Ethane molecules can only occupy the large cavities (5^12^6^2^) of the sI hydrate lattice, while methane can occupy both small (5^12^) and large cavities (5^12^6^2^).

The large cavities become unstable and collapse when the gas molecules escape from them, while some of the small cavities may still remain stable for a certain period of time when methane molecules escape from them^70^. All these factors result in decomposition of double sI hydrate involving the number ratio of large cavities (5^12^6^2^) to small cavities (5^12^) no longer retaining the required value of 3:1 for the stabilization of the sI hydrate lattice. In this case, some of the 5^12^6^2^ cavities can transform into larger ones (5^12^6^4^) to construct a sII lattice with excess small cavities (5^12^). This could be a key feature of the mechanism of structure transition which occurs during the decomposition of double methane-ethane hydrate. Dec *et al*.^45^ used ^13^C MAS NMR to study the decomposition of methane + ethane structure I hydrate. Although the sI/sII transition was not detected due to the different temperature conditions, the NMR resonances also clearly demonstrate that C_2_H_6_ sI large cages decompose more readily than CH_4_ sI small cages.

**Figure 10:**
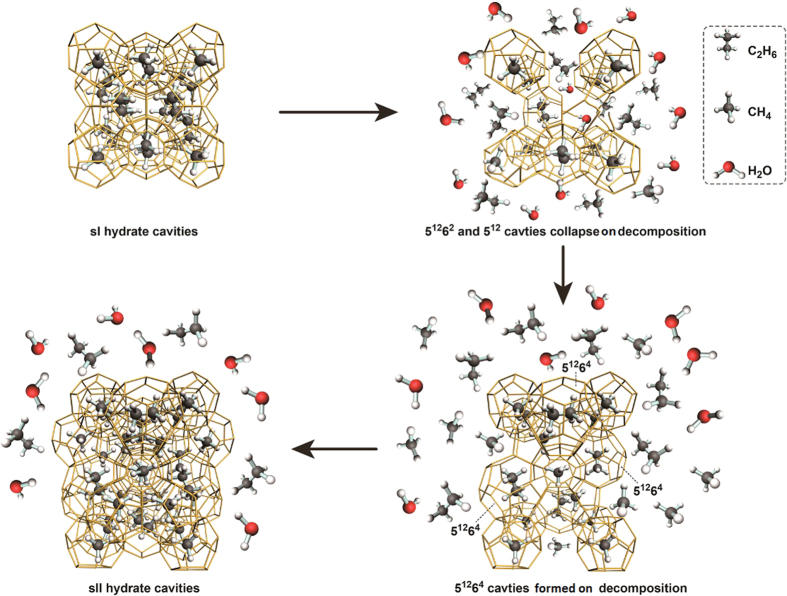
Schematic diagram for the dissociation process of methane-ethane double hydrate with the elapsed time.

In the third stage, methane-ethane hydrate converts completely into sII hydrate. As seen from Fig. 9, at 271.15 K, 270.15 K and 269.15 K the 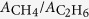 value still increases for a long time period, even after the completion of the structure transition from sI to sII. It seems that self-preservation is selective, i.e., methane shows a stronger self-preservation effect than ethane. To illustrate the mechanism of hydrate dissociation, the schematic diagram for the dissociation process of methane-ethane double hydrate with the elapsed time is shown in Fig. 10.

In summary, the dissociation behavior of pure methane hydrate and methane-ethane double hydrate at temperatures ranging from 272.15 to 268.15 K and atmospheric pressure has been investigated systematically via *in situ* Raman spectroscopy. The sI methane hydrate shows a significant self-preservation effect during dissociation at temperatures from 268.15 to 270.15 K, which has been verified by the C-H peak position and the variation of the *A*_L_/*A*_S_ value, and leads to the stability of methane hydrate at these temperatures. However, the self-preservation effect is weak at higher temperatures (≥271.15 K), as shown by the dramatic change in the spectra and fluctuations of *A*_L_/*A*_S_ values for methane hydrate.

The self-preservation effect also occurs at lower temperature (lower than 271.15 K) for methane-ethane hydrate. The structure transition from sI to sII occurred during the decomposition process of methane-ethane hydrate when at 271.15 K, 270.15 K, and 269.15 K, as identified by the shift in C-H peak position and the variation of the *A*_L_/*A*_S_ value. The values of *A*_L_/*A*_S_ decrease continuously from around 3 and gradually stabilized at 0.7 to 1.2. The mechanism of the structural transition was further studied by the analysis of the variation of *A*_L_/*A*_S_ and 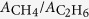 values. The results suggest that the selective self-preservation effect of methane over ethane leads to the structure transition; and this kind of selectivity increases with decreasing temperature.

This work presents the important influence of the self-preservation effect on the stability of methane hydrate and structural transition of methane-ethane double hydrate during dissociation at temperatures below the ice point. The new findings obtained in this work should be important to improve our understanding of the kinetic behavior of hydrate dissociation below the ice point. This information can be helpful for future investigations of the mechanism of hydrate formation or dissociation by Raman spectroscopy, and could help to provide important insight for the further development of gas storage by hydrates.

## Methods

### Procedures

Methane and ethane, both with a purity of 99.9 mol.% (supplied by Beifen Gas Industry Corporation, China), were used in the preparation of the gas mixture and formation of hydrates. Sodium dodecyl sulfate (SDS, analytical reagent) was commercially available (supplied by Beijing Reagents Corporation, China) and used without further purification. Deionized (DI) water was used to prepare hydrates.

**Figure 11:**
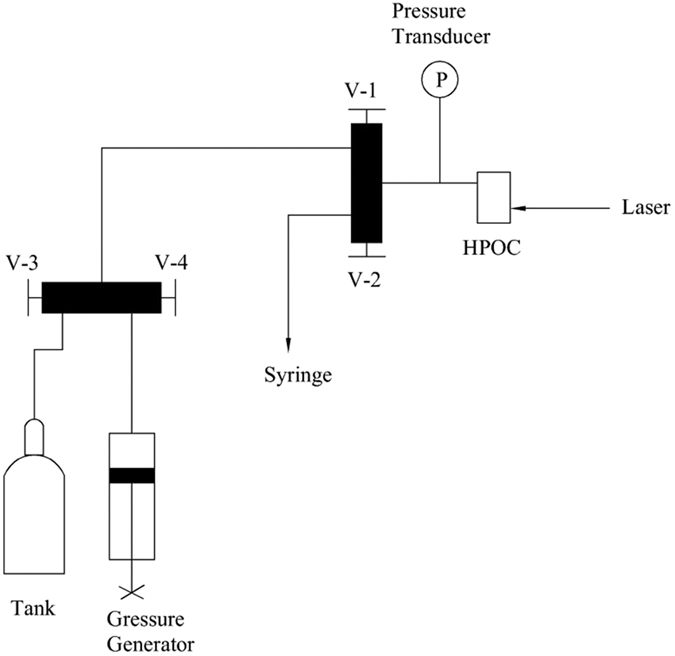
Schematic diagram of the experimental apparatus.

Figure 11 shows the schematic diagram of the experimental apparatus used for the *in situ* Raman spectroscopy study of hydrate samples. Taking methane hydrate formation and dissociation for example, the procedures are presented as follows: (1) load the sample cell half full of 0.5 mL 1000 mg/L SDS aqueous solution or deionized water, in which SDS could accelerate the hydrate formation process^71^,^72^,^73^; (2) place the HPOC on X-Y translational stage of Raman analyzers and focus the Raman laser source on sample; (3) slowly cool the HPOC to 276.15 K and maintain for 2 h; (4) evacuate and flush the sample cell and gas line with gas, which was prepared for hydrate formation: (a) keep the cylinder valve closed, open V-1, V-2, V-3 and V-4, and use the syringe to evacuate the line; (b) close V-2 and open the cylinder valve to fill the HPOC and gas line with gas; (c) open V-2 to flush the line with the sample fluid for 2 minutes; (d) close V-2; (5) close V-3 and pressurize the system to 5 MPa using the pressure generator; (6) maintain the system pressure at 5 MPa for 24 h to allow the gas to fully dissolve in the solution and until the hydrate is formed; (7) slowly cool the HPOC to a temperature below the ice point and maintain at this temperature for 2 h; (8) open V-2 to slowly decrease the pressure to equilibrium pressure corresponding to the current temperature, then quickly lower the system pressure to atmospheric pressure; (9) when the system pressure was reduced to atmospheric pressure, the hydrate dissociation process started and was monitored by Raman spectroscopy. (10) Change of the initial decomposition temperature of hydrate in step (7) and repeat (7)–(9). It should be noted that a large number of such experiments were repeated and every experiment was conducted on different interface spots.

**Figure 12:**
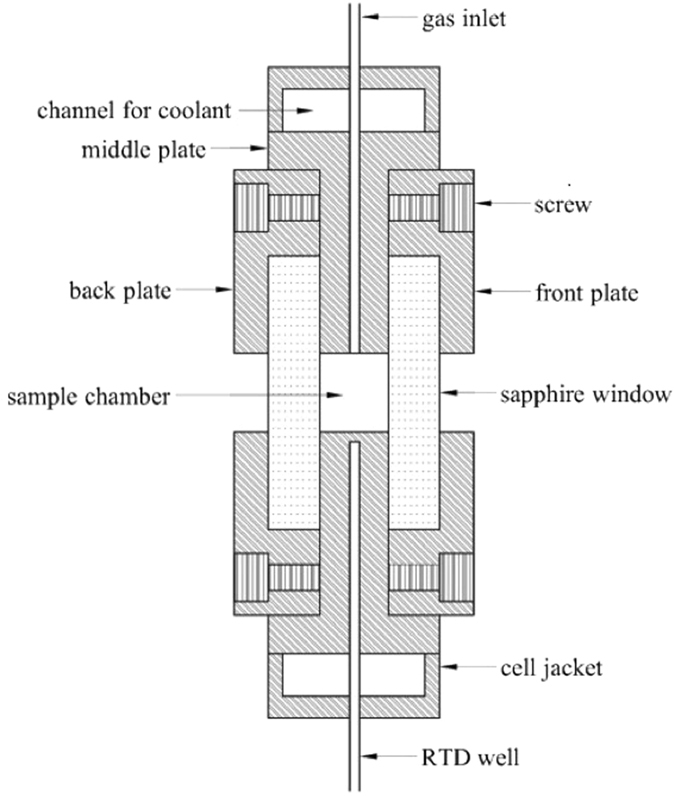
View of the HPOC.

In these experiments, the laser passed through the sapphire window and focused on hydrate as shown in Fig. 12. Moreover, the cell was affixed to an X–Y–Z translational stage so that the laser can focus on a specific hydrate position. It should be noted that the experiments were repeated many times and measurement was conducted on different spots of the hydrate.

### High pressure optical cell (HPOC)

A stainless steel cell similar to Subramanian *et al*.^38^,^74^ was designed for safe operation at pressures of up to 80 MPa, as illustrated in Fig. 12. The whole HPOC is about 115 mm in diameter and 35 mm in height, and constructed from three major parts, which are fixed together by screws: the front plate, the middle plate, and the back plate. The built-in channel in the HPOC is designed to circulate coolant and control temperature. A temperature sensor inserted through a RTD well is placed only a few millimeters from the sample chamber bottom. One gas inlet in the middle of the sample cell wall allows the sample gas to enter the sample chamber and connects with a pressure sensor. The sample cell is 1.4 mL in volume and has two sapphire windows (6 mm in thickness).

### Raman

The Raman spectra were obtained on a HORIBA XploRA Raman system equipped with a 1800 grooves/mm grating and 20× microscope objective. A 532 nm wavelength laser, with excitation power of 20 mW, was used to focus on a fixed middle spot of the hydrate surface in all experiments, and the focused diameter was approximately 5 μm. The spectral resolution was 1 cm^−1^. Routine calibration of the monochromator was performed by using single crystal silicon. The data acquisition time for one measurement was about 180 s, and the exposure time was 60 s for each accumulation. The spectra were averaged over 3 accumulations. The collected Raman spectra were analyzed using Labspec 5 spectral analysis software, in which deconvolution of peaks, peak-fitting, and calculation of the peak areas were performed (see Supporting Information). The peak-fitting parameters were the same for all series of experiments. Initially, the deionized water is loaded inside the HPOC for a week under the experimental pressure to ensure full gas saturation. The formation of hydrate and Raman measurement of hydrate dissociation requires two days. Approximately 140 independent Raman spectra are acquired in one experiment. These spectra were collected on different locations of the hydrate sample.

### Quantitative analysis of Raman spectroscopy

The quantitative analysis of Raman spectral data is based on Placzek’s polarizability theory^75^,^76^,^77^ as given by


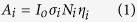


where *A*_*i*_ is the integrated peak area (intensity) of a Raman active peak for the guest species *i* over a finite range, *I*_*o*_ is the irradiance on the sample, *σ*_*i*_ is the Raman scattering cross section of *i* at a certain wavelength of the exciting source, *N*_*i*_ is the number of guest molecules *i* in the irradiated volume, η_*i*_ is the instrumental efficiency of the optical and electronic response^78^. When assuming *I*_*o*_, *σ*_*i*_ and η_*i*_ of different guest species in hydrate lattices being identical^76^, the following equation can be used to evaluate the relative proportion of two guest species *a* and *b* trapped in hydrate lattices.


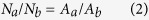


This equation can be used to evaluate the relative proportion of different guest molecules encaged in the hydrate lattice. Similarly, we can also evaluate the relative proportion of occupancies of guest molecules in two different types of cavities by


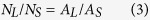


where *N*_L_ and *N*_S_ are the numbers of guest molecules trapped in large and small cavities, respectively; *A*_L_ and *A*_S_ denote the peak area corresponding to the occupancies of guest molecules in large and small cavities of hydrate lattices, respectively.

## Additional Information

**How to cite this article:** Zhong, J.-R. *et al*. Self-preservation and structural transition of gas hydrates during dissociation below the ice point: an *in situ* study using Raman spectroscopy. *Sci. Rep.* **6**, 38855; doi: 10.1038/srep38855 (2016).

**Publisher's note:** Springer Nature remains neutral with regard to jurisdictional claims in published maps and institutional affiliations.

## Supplementary Material

Supplementary Information

## Figures and Tables

**Table 1:** Assignments and peak positions of C-H region Raman spectra for CH_4_ and C_2_H_6_ in sI and sII hydrate lattices.

Guest	Vibrational modes	Peak position (cm^−1^)			
		sI (5^12^)	sI (5^12^6^2^)	sII (5^12^)	sII (5^12^6^4^)
CH_4_ (C1)	Perturbed *v*_1_ symmetric C-H stretch	2914	2904	2913	2903
C_2_H_6_ (C2)	Perturbed *v*_1_ symmetric C-H stretch	—	2891	—	2887
	Perturbed overtone mode of one of the CH_3_ deformation vibrations (2*v*_11_)	—	2946	—	2942
