# Supplementary material for: Self-preservation and structural transition of gas hydrates during dissociation below the ice point: an in situ study using Raman spectroscopy
Source: Sci Rep. 2016 Dec 12;6:38855. doi: 10.1038/srep38855 (PMC5150642; doi:10.1038/srep38855)
Supplement: Supplementary Information [file srep38855-s1.pdf]

Self-preservation and structural transition of gas hydrates during dissociation below the ice point: an *in situ* study using Raman spectroscopy

Jin-Rong Zhong [1,†], Xin-Yang Zeng[1,†], Feng-He Zhou[1], Qi-Dong Ran[1], Chang-Yu Sun[1,*], Rui-Qin Zhong[1,*],Lan-Ying Yang[1], Guang-Jin Chen[1], Carolyn A. Koh[2]

[1]State Key Laboratory of Heavy Oil Processing, China University of Petroleum, Beijing, 102249, China

[2]Center for Hydrate Research, Colorado School of Mines, Golden, Colorado 80401, United States

[*] To whom correspondence should be addressed. Fax: +86 10 89733156. E-mail: cysun@cup.edu.cn (C.Y. Sun); zhong2004@hotmail.com (R.Q. Zhong)

[†] These authors contributed equally to this work.

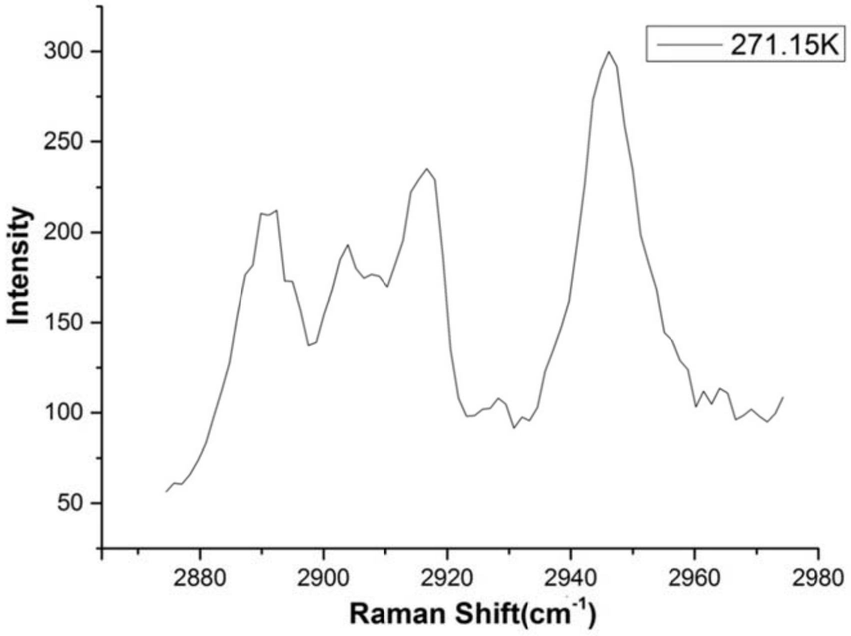

Figure S1. Original Raman spectra collected by Labspec 5 at 271.15 K and 31 min for the dissociation of methane-ethane hydrate.

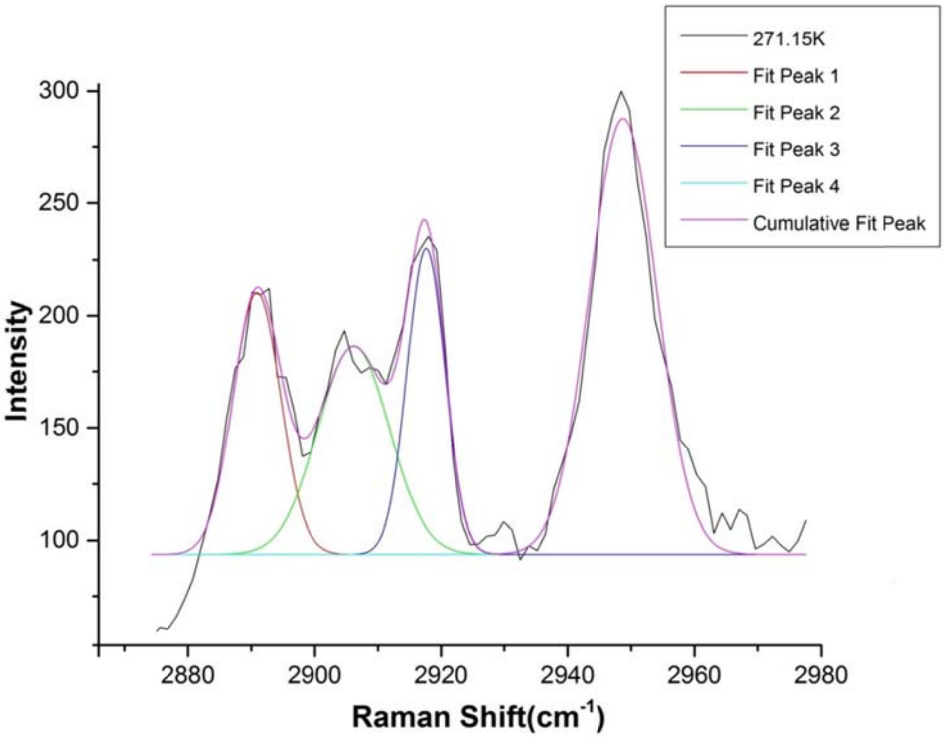

Figure S2. Peak-fitting for Raman spectra collected by Labspec 5 at 271.15 K and 31 min for the dissociation of methane-ethane hydrate
